# Supplementary material for: Reprogramming biocatalytic futile cycles through computational engineering of stereochemical promiscuity to create an amine racemase
Source: Nat Commun. 2024 Jan 2;15:49. doi: 10.1038/s41467-023-44218-7 (PMC10761954; doi:10.1038/s41467-023-44218-7)
Supplement: Supplementary file 1 — Supplementary Information [file 41467_2023_44218_MOESM1_ESM.pdf]

Supplementary Information for:

Reprogramming biocatalytic futile cycles through computational engineering of stereochemical promiscuity to create an amine racemase

Sang-Woo Han[1,2], Youngho Jang[1], Jihyun Kook[1], Jeesu Jang[1], and Jong-Shik Shin[1,*]

[1] Department of Biotechnology, Yonsei University, 50 Yonsei-Ro, Seodaemun-Gu, Seoul 03722, South Korea

[2] Present address: Department of Biotechnology, Konkuk University, Chungju, South Korea

* To whom correspondence should be addressed. e-mail: enzymo@yonsei.ac.kr

Table of Contents

Supplementary Methods……………………………………………………………………..………..3

Supplementary Tables……………………………………………………………………….....6

Table 1. Enzyme activities of the alanine scanning mutants of ATA-OA for *R*-**D1**.
Table 2. Conservation of the F86* residue among *S*-selective ATAs.
Table 3. Sequences of the mutagenesis primers.
Table 4. Reaction conditions and product analysis for the kinetic measurements to determine k_{OD} and k_{RA}.
Table 5. Chiral HPLC analysis conditions.

Supplementary Figures…………………………………………………….…..……11

Figure 1. Resonance structure stabilization of the carbanionic intermediate owing to the enhanced electron withdrawing capability of the protonated pyridine of PLP.
Figure 2. Time-course monitoring of the chiral composition of **D1** during the ATA-OA reactions.
Figure 3. Time-course monitoring of ee^S of the **D2** product during the reactions shown in Fig. 2c.
Figure 4. Time-course monitoring of the chiral composition of the 3-methyl-2-butylamine product during the reaction between 3-methyl-2-butanone and **D4** by ATA-OA.
Figure 5. Typical activity measurements with *S*-**D1** and *R*-**D1** for kinetic analysis of ATA-OA.
Figure 6. Effect of the substrate concentration on the initial rate of II^S/II^R and II^R/II^S.
Figure 7. Effect of DMSO on the racemization of *S*-**D2** and *R*-**D2** by ATA-OA.
Figure 8. Numerical simulations of the I^S/I^R and I^R/I^S reactions catalyzed by a hypothetical ATA-OA with tunable E^S.
Figure 9. All docking poses of *R*-**D1** in ATA-OA.
Figure 10. The top docking pose of *S*-**D1** (thick sticks) in the F86*A mutant relative to that (thin magenta sticks) in the wild type.
Figure 11. Reduced stereoselectivity of ATA-PD for **D1** caused by the F85*A mutation.
Figure 12. Activities of the F86*L mutant of ATA-OA for *S*-**D1** and *R*-**D1** in comparison with those of the wild type.
Figure 13. Wavelength scanning of AR-OA equilibrated with *S*-**D1** or *R*-**D1**.
Figure 14. Racemization of *S*-**D2** by AR-OA in comparison with that by ATA-OA.
Figure 15. Effect of the **A1** concentration on the initial rate of I^S/I^R by AR-OA.
Figure 16. Cosubstrate-free racemization of *S*-**D1** by AR-OA.
Figure 17. Structural characterizations of the isolated *rac*-**D5**.
Figure 18. Racemization of L- and D-alanine by the ATA-OA mutant carrying R417A and W58L substitutions.

Supplementary References……………….…………….……………………………29

Supplementary Methods

Chemicals and Materials. All chemicals purchased from commercial sources were of the highest purity available and used without further purification. Enantiopure amines of **D6**, **D10**, and **D16-D18** were purchased from Alfa Aesar. *Rac*-**D17** was obtained from Tokyo Chemical Industry. Enantiopure **D5** and L-alanine were purchased from Acros Organics. **D8** was obtained from Junsei Chemical Co. All other chemicals were purchased from Sigma-Aldrich. Materials used for preparation of cell culture media, including yeast extract, tryptone and agar, were purchased from Difco.

Mathematical formulation of the second-order rate equation for kinetic analysis. The ATA reaction comprises two half reactions, OD and RA. To determine the rate constant for a specific half reaction, it is necessary for the other half reaction to proceed much faster than the one being observed. In the kinetic analysis, we achieved this using a reactive cosubstrate at a concentration higher than its K_M, while keeping the substrate concentration much lower than the corresponding K_M. As a result, the kinetic measurements for the complete reaction were approximated to the half reaction rate that we intended to observe.

Based on the ping-pong bi-bi mechanism of transaminases, the initial rate (v_i) under the negligible product formation is expressed by

$$v_i = \frac{V_{max}[A][D]}{K_M^D[A]+K_M^A[D]+[A][D]} \quad (1)$$

where K_M^D and K_M^A represent the Michaelis constants for the donor and acceptor, respectively. To derive a second-order rate equation to determine k_{OD}, Eq. 1 is first modified to

$$v_i = \frac{V_{max}[D]}{K_M^D+\left(1+\frac{K_M^A}{[A]}\right)[D]} \quad (2)$$

by dividing the numerator and denominator of the fraction by [A]. The cosubstrate we used to determine k_{OD} of **D1** and **D2** was pyruvate for which K_M of ATA-OA was determined to be 1.9 mM.[1] We estimate that using a pyruvate concentration much higher than its K_M cancels out the $K_M^A/[A]$ term in Eq. 2. Therefore, we used 100 mM pyruvate for the kinetic measurements. Under the cosubstrate conditions, Eq. 2 can be simplified to a single-substrate Michaelis-Menten kinetics as follows.

$$v_i \approx \frac{V_{max}[D]}{K_M^D+[D]} \quad (3)$$

Eq. 3 is further simplified to

$$v_i \approx \frac{V_{max}}{K_M^D}[D] = \frac{k_{cat}}{K_M^D}[E]_0[D] \quad (4)$$

where $[E]_0$ represents the total enzyme concentration, under the reaction conditions of $[D] \ll K_M^D$. For example, $K_M^{S-\mathbf{D1}}$ of ATA-OA was determined to be 150 mM.[2] Thus, we used 10 mM D for the kinetic measurements, which is a reasonable reaction condition to meet $[D] \ll K_M^D$. The purified enzyme consists of the E-PLP and E-PMP forms. However, under the reaction conditions where $[\text{pyruvate}]/K_M^{\text{pyruvate}} \gg [D]/K_M^D$, it can be approximated that the enzyme is mostly present as a E-PLP form, i.e. $[E]_0 \approx [\text{E-PLP}]$. Therefore, Eq. 4 can be developed as follows by defining k_{OD} as a specificity constant for D.

$$v_i \approx \frac{k_{cat}}{K_{\mathrm{M}}^{\mathrm{D}}}[\mathrm{E-PLP}][\mathrm{D}] = k_{\mathrm{OD}}[\mathrm{E-PLP}][\mathrm{D}] \quad (5)$$

Eq. 5 underlies the second-order kinetic approximation for the half reaction. As a result, k_{OD} can be determined by the kinetic measurements using Eq. 6.

$$k_{\mathrm{OD}} \approx v_i/([\mathrm{E}]_0[\mathrm{D}]) \quad (6)$$

Likewise, the expression for k_{RA} can be derived following the similar procedures. Dividing the numerator and denominator of the fraction in Eq. 1 by [D] leads to Eq. 7.

$$v_i = \frac{V_{max}[\mathrm{A}]}{K_{\mathrm{M}}^{\mathrm{A}} + \left(1 + \frac{K_{\mathrm{M}}^{\mathrm{D}}}{[\mathrm{D}]}\right)[\mathrm{A}]} \quad (7)$$

The cosubstrate used to determine k_{RA} of **A1** and **A2** was **D8** for which K_{M} of ATA-OA was determined to be 400 mM.[1] The $K_{\mathrm{M}}^{\mathbf{D8}}$ value is rather high to set the reaction conditions for $[\mathrm{D}] \gg K_{\mathrm{M}}^{\mathrm{D}}$. As a compromise, we used 500 mM **D8** and the resulting $1 + K_{\mathrm{M}}^{\mathrm{D}}/[\mathrm{D}]$ was 1.8. We estimated that using [A] much lower than $K_{\mathrm{M}}^{\mathrm{A}}$ can allow us to ignore the $(1 + K_{\mathrm{M}}^{\mathrm{D}}/[\mathrm{D}])[\mathrm{A}]$ term in Eq. S7. The $K_{\mathrm{M}}^{\mathbf{A1}}$ and $K_{\mathrm{M}}^{\mathbf{A2}}$ values were determined to be 110 mM[2] and 4000 mM,[1] respectively. Therefore, we ignored the $(1 + K_{\mathrm{M}}^{\mathrm{D}}/[\mathrm{D}])[\mathrm{A}]$ term by accepting 16 and 0.4 % approximation errors of the kinetic measurements for 10 mM **A1** and **A2**, respectively. Based on the reasoning, Eq. 7 was approximated to

$$v_i \approx \frac{k_{cat}}{K_{\mathrm{M}}^{\mathrm{A}}}[\mathrm{E}]_0[\mathrm{A}]. \quad (8)$$

Considering $[\mathbf{D8}]/K_{\mathrm{M}}^{\mathbf{D8}} \gg [\mathrm{A}]/K_{\mathrm{M}}^{\mathrm{A}}$ under the reaction conditions, we approximated that the enzyme is mostly present as a E-PMP form, i.e. $[\mathrm{E}]_0 \approx [\mathrm{E\text{-}PMP}]$. Therefore, Eq. 8 can be expressed as follows by defining k_{RA} as a specificity constant for A.

$$v_i \approx \frac{k_{cat}}{K_{\mathrm{M}}^{\mathrm{A}}}[\mathrm{E-PMP}][\mathrm{A}] = k_{\mathrm{RA}}[\mathrm{E-PMP}][\mathrm{A}] \quad (9)$$

As a result, we determined k_{RA} using Eq. 10.

$$k_{\mathrm{RA}} \approx v_i/([\mathrm{E}]_0[\mathrm{A}]) \quad (10)$$

Cell cultivation and enzyme purification. *Escherichia coli* BL21(DE3) cells transformed with the pET28a(+) expression vectors were cultivated in LB medium (typically 1 L) containing 50 μg/mL kanamycin at 37 °C. Protein overexpression was induced by IPTG (final concentration = 0.1 mM) at $OD_{600} \approx 0.4$, and then cells were cultivated further for 10 h. The culture broth was centrifuged (10,000 × g, 10 min, 4 °C) and the resulting cell pellet was resuspended in 15 mL resuspension buffer (50 mM Tris-HCl, pH 7, 50 mM NaCl, 1 mM EDTA, 1 mM β-mercaptoethanol, 0.1 mM PMSF, 0.02 % sodium azide and 0.5 mM PLP). The cells were disrupted by a sonicator and then centrifuged (13,000 × g, 60 min, 4 °C) to remove cell debris.

The resulting cell-free extract was subjected to protein purification on an ÄKTAprime plus (GE Healthcare). The cell-free extract was loaded on a HisTrap HP column (GE Healthcare) and the His6-tagged ω-TA was eluted by an elution buffer (20 mM sodium phosphate, 0.5 M sodium chloride, 0.5 mM PLP, pH 7.4) with a linear gradient of imidazole (0.02 - 0.5 M). Imidazole was removed by a HiTrap desalting column (GE Healthcare) using an elution buffer (50 mM sodium phosphate, 0.15 M sodium chloride and 0.2 mM PLP, pH 7). When necessary, the enzyme solution was concentrated using an ultrafiltration kit (Ultracel-30) purchased from Millipore Co.

Mathematical modeling of enzyme reactions. For numerical simulations of the reactions involving I^S, II^S, and II^R, balance equations were derived for *S*-**D1**, **A1**, *S*-**D2**, *R*-**D2**, **A2**, E-PLP, and E-PMP.

$$d[S\text{-}\mathbf{D1}]/dt = -k_{OD,I^S}[S\text{-}\mathbf{D1}][\text{E-PLP}] + k_{RA,I^S}[\mathbf{A1}][\text{E-PMP}] \quad (11)$$

$$d[\mathbf{A1}]/dt = k_{OD,I^S}[S\text{-}\mathbf{D1}][\text{E-PLP}] - k_{RA,I^S}[\mathbf{A1}][\text{E-PMP}] \quad (12)$$

$$d[S\text{-}\mathbf{D2}]/dt = -k_{OD,II^S}[S\text{-}\mathbf{D2}][\text{E-PLP}] + k_{RA,II^S}[\mathbf{A2}][\text{E-PMP}] \quad (13)$$

$$d[R\text{-}\mathbf{D2}]/dt = -k_{OD,II^R}[R\text{-}\mathbf{D2}][\text{E-PLP}] + k_{RA,II^R}[\mathbf{A2}][\text{E-PMP}] \quad (14)$$

$$d[\mathbf{A2}]/dt = k_{OD,II^S}[S\text{-}\mathbf{D2}][\text{E-PLP}] - k_{RA,II^S}[\mathbf{A2}][\text{E-PMP}] + k_{OD,II^R}[R\text{-}\mathbf{D2}][\text{E-PLP}] - k_{RA,II^R}[\mathbf{A2}][\text{E-PMP}] \quad (15)$$

$$d[\text{E-PLP}]/dt = -k_{OD,I^S}[S\text{-}\mathbf{D1}][\text{E-PLP}] + k_{RA,I^S}[\mathbf{A1}][\text{E-PMP}] - k_{OD,II^S}[S\text{-}\mathbf{D2}][\text{E-PLP}] + k_{RA,II^S}[\mathbf{A2}][\text{E-PMP}] - k_{OD,II^R}[R\text{-}\mathbf{D2}][\text{E-PLP}] + k_{RA,II^R}[\mathbf{A2}][\text{E-PMP}] \quad (16)$$

$$d[\text{E-PMP}]/dt = k_{OD,I^S}[S\text{-}\mathbf{D1}][\text{E-PLP}] - k_{RA,I^S}[\mathbf{A1}][\text{E-PMP}] + k_{OD,II^S}[S\text{-}\mathbf{D2}][\text{E-PLP}] - k_{RA,II^S}[\mathbf{A2}][\text{E-PMP}] + k_{OD,II^R}[R\text{-}\mathbf{D2}][\text{E-PLP}] - k_{RA,II^R}[\mathbf{A2}][\text{E-PMP}] \quad (17)$$

The seven differential equations were numerically solved using Mathematica 12 with kinetic parameter values listed in Fig. 2e. In the case of the simulation for Fig. 3a, kinetic parameters for I^S_{RA}, II^S_{OD}, and II^R_{OD} were set to be zero. For simulations of the direct racemization in Fig. 3d, Eq. 11 and 12 were excluded. Since the protein purification was carried out using buffers supplemented with PLP, the enzyme was assumed to be present as an E-PLP form only at the beginning of the simulations. The initial conditions used for simulations in Fig. 3a-3c were 50 mM *S*-**D1**, 50 mM **A2**, and 750 µM E-PLP. Initial concentrations of the other four components were zero. The initial conditions used for the simulation in Fig. 3d were 50 mM *S*-**D2** or *R*-**D2**, 50 mM **A2**, and 100 µM E-PLP.

Chiral analysis of alkylamines and alanine using a Marfey's reagent. In a typical derivatization procedure using a Marfey's reagent, reaction samples were appropriately diluted with acetonitrile (the molar ratio of Marfey's reagent to alkylamine or alanine was 1.4:1), and then 10 µL sample was mixed with 8 µL of 1 M sodium bicarbonate solution and 40 µL of 1 % Marfey's reagent. The reaction mixture was vortexed, heated at 40 ^{0}C for an hour, and cooled to room temperature. Then 8 µL of 1 M hydrochloric acid was added to quench the reaction. The sample was diluted with 434 µL of 40/60 % acetonitrile/water and aliquots of the sample (10 µL) were analyzed with a Waters HPLC system using a Symmetry C18 column warmed at 40 °C. The eluent flow rate was 1 mL/min and a UV detector was tuned at 320 nm.

Supplementary Tables

Supplementary Table 1. Enzyme activities of the alanine scanning mutants of ATA-OA for *R*-**D1**.[a]

Mutation	Specific activity (U mL^{-1} mM-$enzyme^{-1}$)
Y20A	n.d.[b]
L57A	n.d.[b]
W58A	n.d.[b]
F86*A	0.380 ± 0.004
Y151A	n.d.[b]
V154A	n.d.[b]
I261A	n.d.[b]
F323*A	n.d.[b]
T324*A	n.d.[b]

[a] Reaction conditions for the enzyme assay were 10 mM *R*-**D1** and 10 mM pyruvate in 50 mM potassium phosphate (pH 7) at 37 °C.

[b] n.d.: not detectable. Compared to a 10-min reaction time and less than 1 μM enzyme concentrations used for routine activity assay with *S*-**D1**, the reactions with *R*-**D1** were allowed to run for 6 hr at up to 100 μM enzyme. However, formation of **A1** leveled off at 0.05 mM which was the same as the initial *S*-**D1** impurity in the reaction mixture.

Supplementary Table 2. Conservation of the F86* residue among *S*-selective ATAs.

Microbial source	PDB ID	Amino acid sequence[a]
Ochrobactrum anthropi	5GHF	QMKKLPFYHT**F**SYRSHGPVIDL 86
Paracoccus denitrificans	4GRX	QYDRFPGYHA**F**FGRMSDQTVML 85
Chromobacterium violaceum	4A6T	QMEELPFYNT**F**FKTTHPAVVEL 88
Vibrio fluvialis	3NUI	QYERFPGYHA**F**FGRMSDQTVML 85
Pseudomonas aeruginosa	4B98	QLGTLDYSPG**F**QYG-HPLSFQL 88
Ruegeria pomeroyi	3HMU	QMRELPYYNT**F**FKTTHVPAIAL 91
Pseudomonas sp.	5LH9	QLDELAYYQT**F**DGIAHPRVFDL 82
Virgibacillus pantothenticus	6FYQ	QMKKMAFSSA**F**STFSHEPAIRL 83
Pseudomonas jessenii	6G4D	QFNTLPFYHL**F**SHKSHRPSIEL 86
Halomonas elongata	6GWI	QLEQLPYYNT**F**FKTTHPPAVRL 84
Thermomicrobium roseum	6IO1	QMERVAFVPT**F**FGLASPPTIEL 89
Ruegeria sp. TM1040	3FCR	QARELAYYHS**Y**VGHGTEASITL 87
Mesorhizobium japonicum	3GJU	QAKNLAYYHA**Y**VGHGTEASITL 88
Cereibacter sphaeroides	3I5T	QAMVLPYASP**W**YMA-TSPAARL 90

[a] The aromatic amino acids corresponding to F86* of ATA-OA are represented in bold. The number following the amino acid sequence designates the location of the conserved aromatic amino acid.

Supplementary Table 3. Sequences of the mutagenesis primers.

Parental enzyme	Mutation	Mutagenesis primers[a]
ATA-OA F86*A	L57A	Forward: 5'-CGAAGCGATGTCAGGAGCGTGGAGTGTTGGCGTG-3'<br>Reverse: 5'-CACGCCAACACTCCACGCTCCTGACATCGCTTCG-3'
	W58A	Forward: 5'-GCGATGTCAGGACTGGCGAGTGTTGGCGTGGG-3'<br>Reverse: 5'-CCCACGCCAACACTCGCCAGTCCTGACATCGC-3'
	V154A	Forward: 5'-CGGCTATCACGGTGCGACGATTGCCTCTG-3'<br>Reverse: 5'-CAGAGGCAATCGTCGCACCGTGATAGCCG-3'
	I261A	Forward: 5'-TCTGCTGATCGCCGACGAGGTTGCGTGCGGCTTCGGA-3'<br>Reverse: 5'-TCCGAAGCCGCACGCAACCTCGTCGGCGATCAGCAGA-3'
ATA-OA	F86*L	Forward: 5'-GAAGAAGCTGCCTTTCTACCATACACTGTCCTACCGTTCGC-3'<br>Reverse: 5'-GCGAACGGTAGGACAGTGTATGGTAGAAAGGCAGCTTCTTC-3'
ATA-PD	F85*A	Forward: 5'-CTTTCCCGGCTATCACGCCTTTGCGGGCCGCATGTCC-3'<br>Reverse: 5'-GGACATGCGGCCCGCAAAGGCGTGATAGCCGGGAAAG-3'

[a] The mutation sites are underlined.

Supplementary Table 4. Reaction conditions and product analysis for the kinetic measurements to determine k_{OD} and k_{RA}.

Enzyme	k	Substrate (conc. in mM)	Cosubstrate (conc. in mM)	[Enzyme] (μM)	Product to be analyzed
ATA-OA	k_{OD}	*S*-**D1** (10)	pyruvate (100)	1	**A1**
		R-**D1** (10)		10	**A1**
		S-**D2** (10)		1	L-alanine
		R-**D2** (10)		10	L-alanine
	k_{RA}	**A1** (10)	**D8** (500)	125	*S*-**D1** and *R*-**D1**
		**A2** (10)		20	*S*-**D2** and *R*-**D2**
AR-OA	k_{OD}	*S*-**D1** (10)	pyruvate (100)	2	**A1**
		R-**D1** (10)		2	**A1**
		S-**D2** (10)		10	L-alanine
		R-**D2** (10)		10	L-alanine
	k_{RA}	**A1** (10)	**D8** (100)	100	*S*-**D1** and *R*-**D1**
		**A2** (10)		125	*S*-**D2** and *R*-**D2**

Supplementary Table 5. Chiral HPLC analysis conditions.

Analyte	Column	Elution conditions[a]	Retention time (min)		UV detection
			R-form	*S*-form	
**D1**	Crownpak CR(-)	water (perchloric acid, pH 2)	13.5	16.8	200 nm
**D2**	Symmetry C18	50/50% (v/v) MeOH/water[b]	37.1	41.2	340 nm
**D3**	Crownpak CR(-)	water (perchloric acid, pH 2)	28.0	36.2	200 nm
**D5**	Symmetry C18	80/20% (v/v) MeOH/water[b]	5.0	5.6	340 nm
**D6**	Crownpak CR(-)	water (perchloric acid, pH 2)	17.5	22.4	200 nm
**D10**	Symmetry C18	80/20% (v/v) MeOH/water[b]	5.6	6.8	340 nm
**D11**	Symmetry C18	80/20% (v/v) MeOH/water[b]	4.3	5.3	340 nm
**D12**	Crownpak CR(-)	water (perchloric acid, pH 2)	29.3	41.0	200 nm
**D13**	Crownpak CR(-)	water (perchloric acid, pH 2)	31.2	37.8	200 nm
**D14**	Crownpak CR(-)	water (perchloric acid, pH 2)	7.5	6.5	200 nm
**D15**	Symmetry C18	70/30% (v/v) MeOH/water[b]	11.9	17.7	340 nm
**D16**	Symmetry C18	70/30% (v/v) MeOH/water[b]	11.9	14.4	340 nm
**D17**	Symmetry C18	80/20% (v/v) MeOH/water[b]	8.4	10.1	340 nm
**D18**	Symmetry C18	40/60% (v/v) MeOH/water[b]	43.3	46.0	340 nm

[a] Flow rate was 0.8 mL/min.

[b] Both MeOH and water contained 0.1 % (v/v) trifluoroacetic acid.

Supplementary Figures

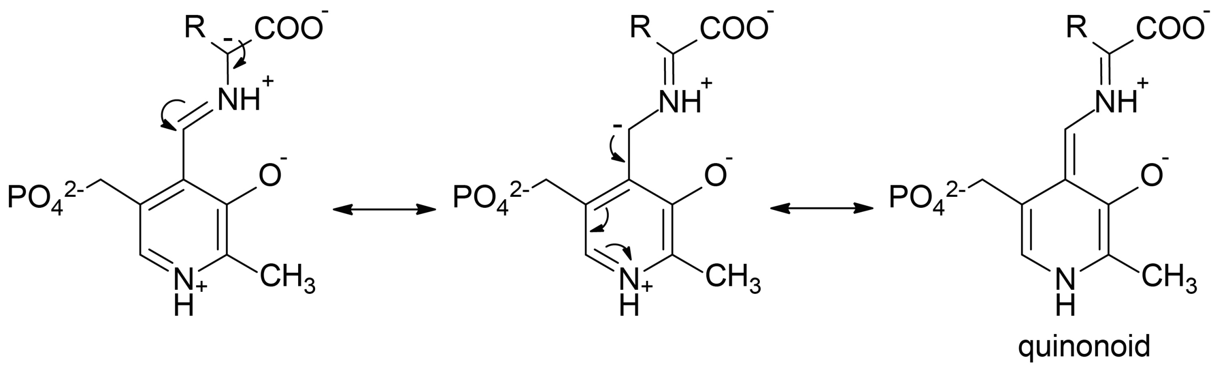

Supplementary Figure 1. Resonance structure stabilization of the carbanionic intermediate owing to the enhanced electron withdrawing capability of the protonated pyridine of PLP. The quinonoid structure is the most stable one among the resonance structures.

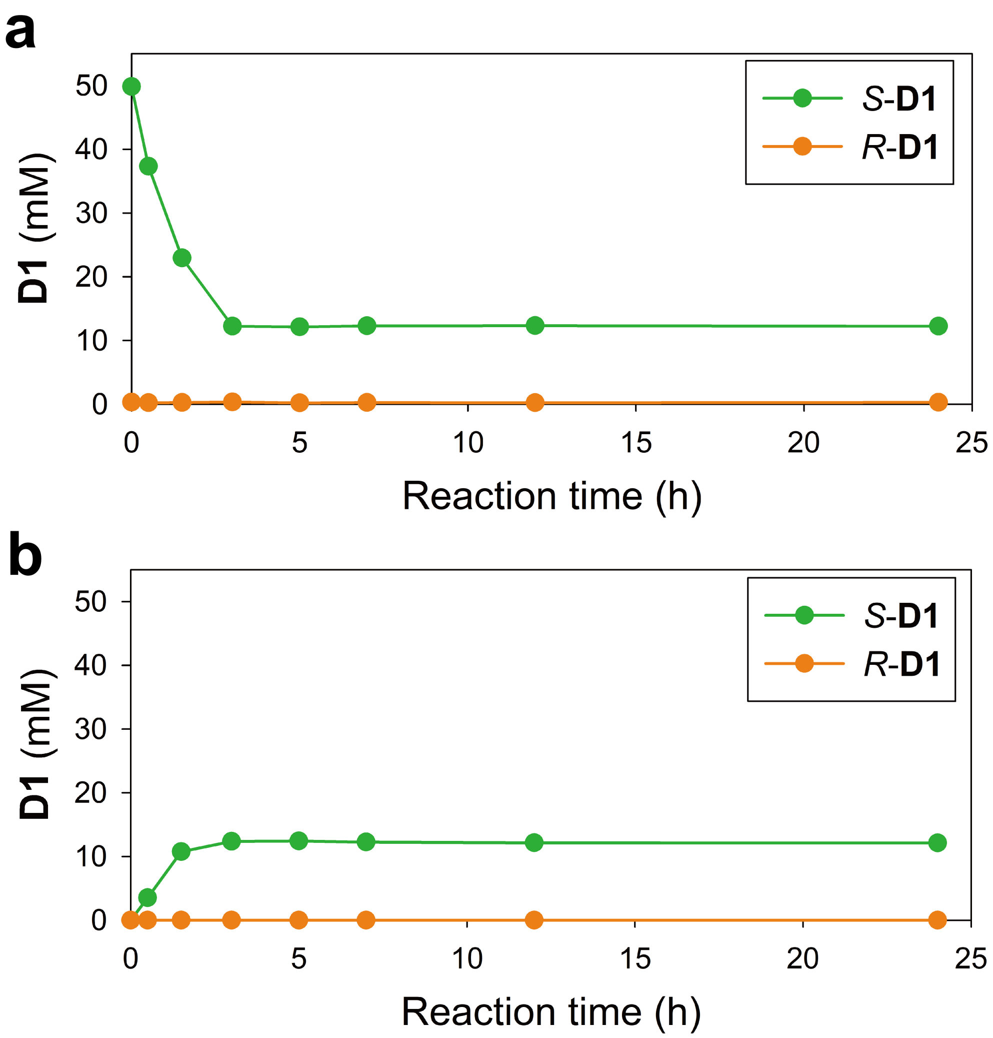

Supplementary Figure 2. Time-course monitoring of the chiral composition of **D1** during the ATA-OA reactions. **a**, Chiral analysis of the *S*-**D1** substrate during the reaction shown in Fig. 2a. **b**, Chiral analysis of the *S*-**D1** product during the reaction shown in Fig. 2b. Source data are provided as a Source Data file.

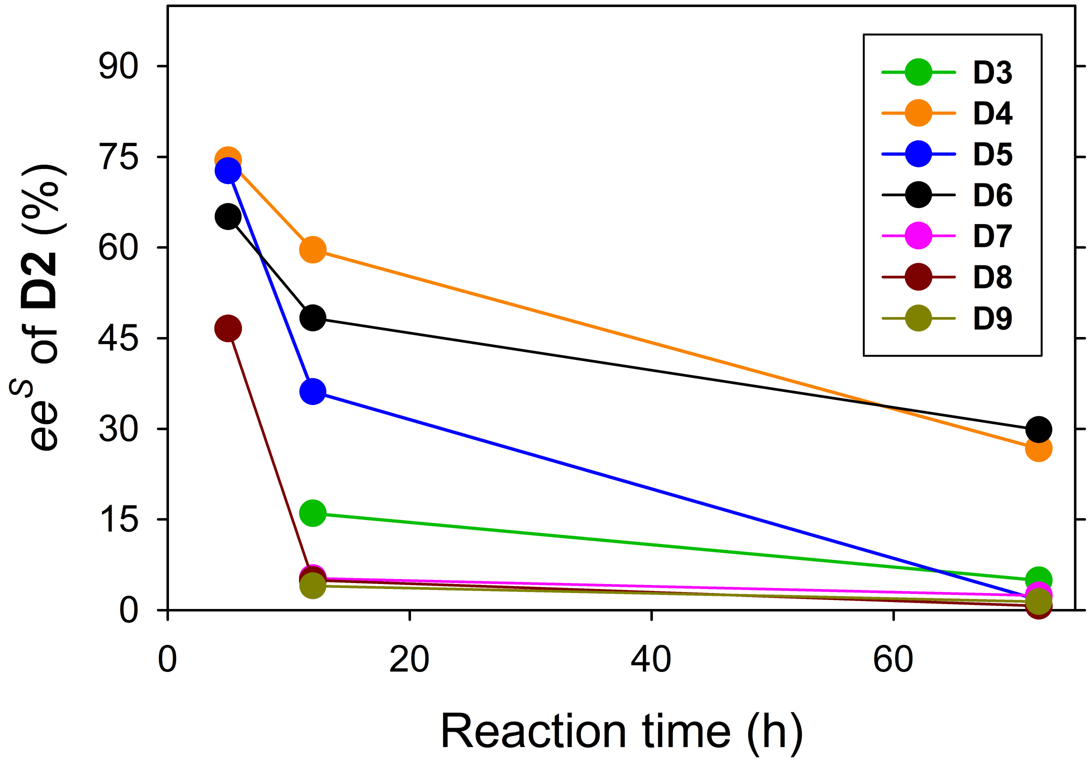

Supplementary Figure 3. Time-course monitoring of *ee*^{S} of the **D2** product during the reactions shown in Fig. 2c. Source data are provided as a Source Data file.

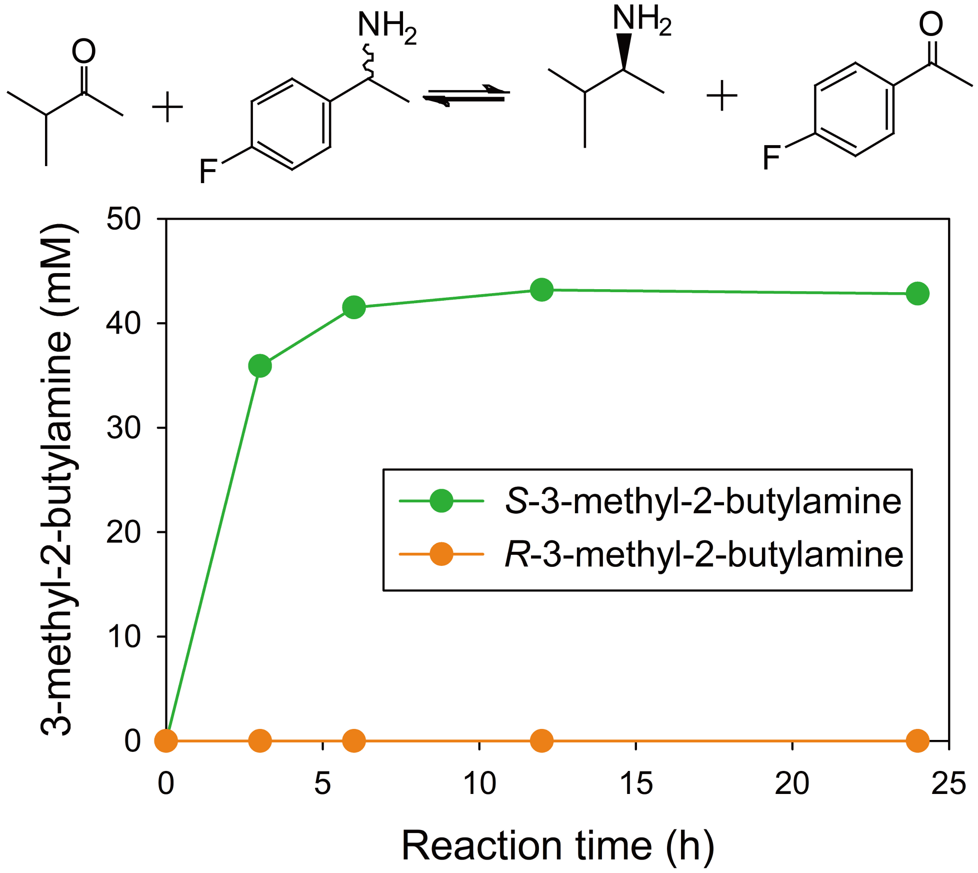

Supplementary Figure 4. Time-course monitoring of the chiral composition of the 3-methyl-2-butylamine product during the reaction between 3-methyl-2-butanone and **D4** by ATA-OA. Reaction conditions were 60 mM 3-methyl-2-butanone, 100 mM *rac*-**D4**, 0.5 mM PLP, 15 % (v/v) DMSO, and 200 U/mL ATA-OA. Source data are provided as a Source Data file.

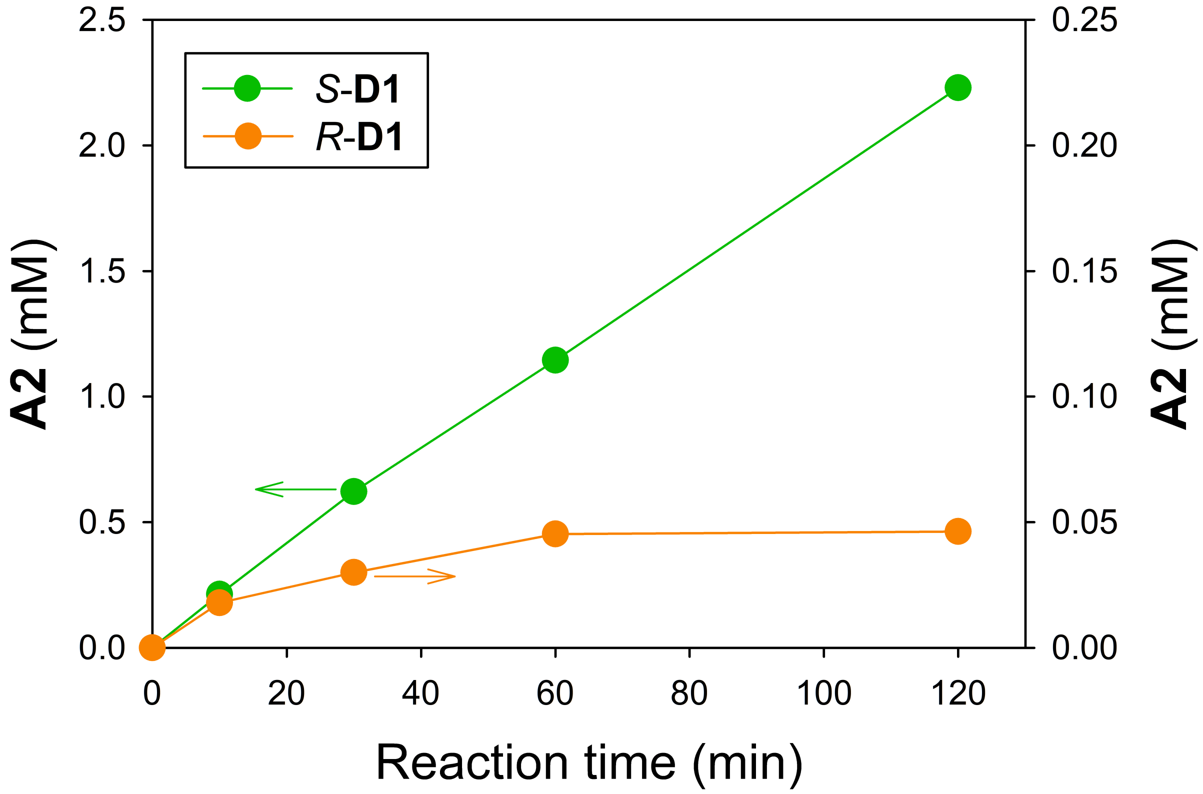

Supplementary Figure 5. Typical activity measurements with *S*-**D1** and *R*-**D1** for kinetic analysis of ATA-OA. Reaction conditions were 10 mM *S*-**D1** or *R*-**D1** and 100 mM pyruvate. Enzyme concentration was 1 and 10 µM for *S*-**D1** and *R*-**D1**, respectively. Note a ten-fold difference in scale between the left and right y-axis. Source data are provided as a Source Data file.

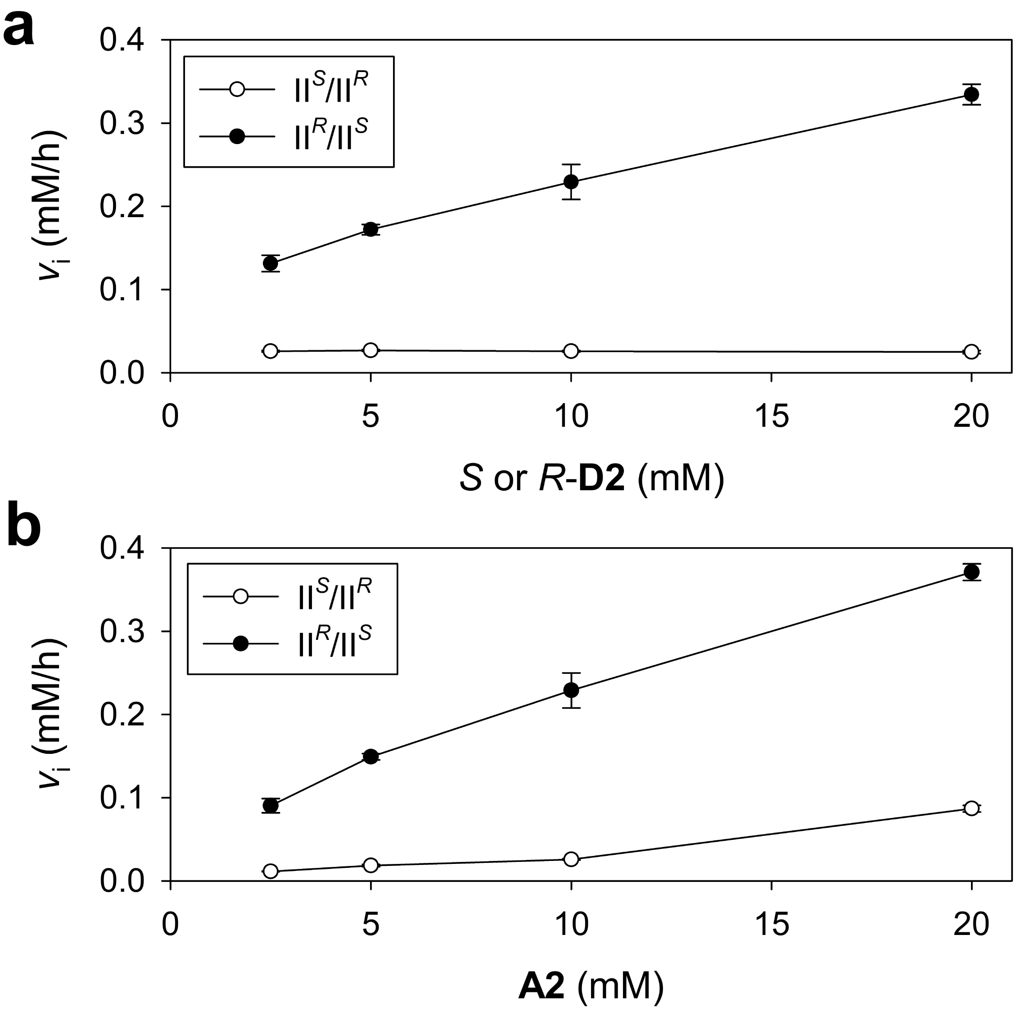

Supplementary Figure 6. Effect of the substrate concentration on the initial rate of II^S/II^R and II^R/II^S. **a**, Dependency of II^S/II^R and II^R/II^S on the concentration of *S*-**D2** and *R*-**D2**, respectively. Reaction conditions were 2.5-20 mM *S*-**D2** or *R*-**D2**, 10 mM **A2**, 0.5 mM PLP, and 50 µM ATA-OA. **b**, Dependency of the initial rate on the concentration of **A2**. Reaction conditions were 10 mM *S*-**D2** or *R*-**D2**, 2.5-20 mM **A2**, 0.5 mM PLP, and 50 µM ATA-OA. Source data are provided as a Source Data file. Data are mean values of triplicate experiments with error bars indicating the s. d. (n = 3).

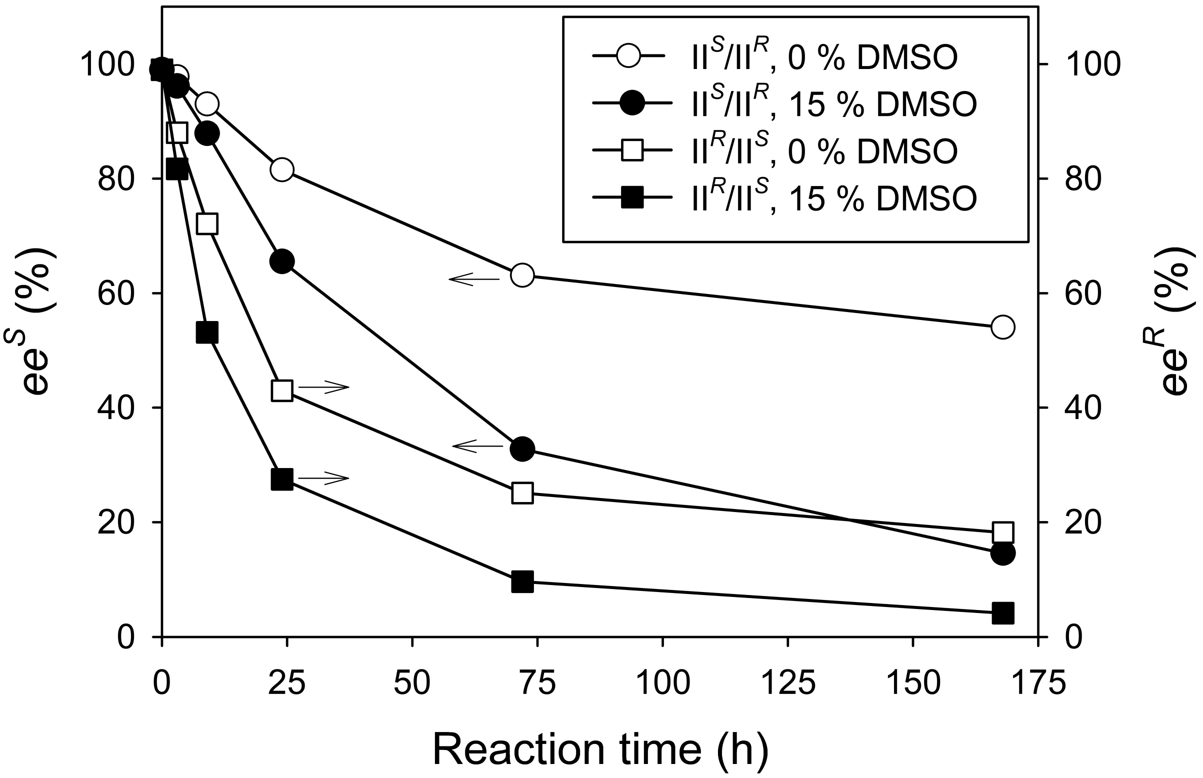

Supplementary Figure 7. Effect of DMSO on the racemization of *S*-**D2** and *R*-**D2** by ATA-OA. Reaction conditions were 10 mM *S*-**D2** or *R*-**D2**, 10 mM **A2**, 0 or 15 % (v/v) DMSO, and 50 μM ATA-OA. Source data are provided as a Source Data file.

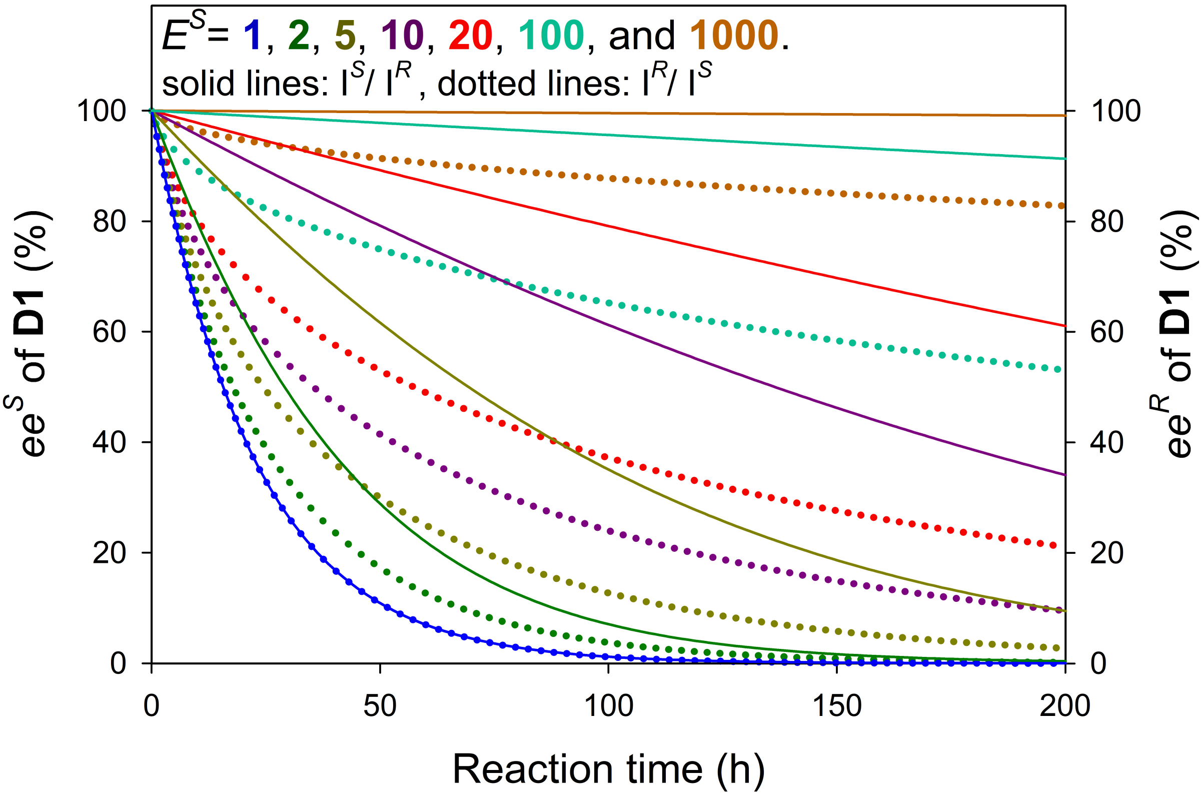

Supplementary Figure 8. Numerical simulations of the I^S/I^R and I^R/I^S reactions catalyzed by a hypothetical ATA-OA with tunable E^S. The k_{OD} and k_{RA} values for I^S used in the simulations were adopted from Fig. 2e and were set to be constant irrespective of E^S. In contrast, the k_{OD} and k_{RA} values for I^R were changed depending on E^S, i.e. $k_{I^R} = k_{I^S}/E^S$. Simulation conditions were 50 mM *S*-**D1** or *R*-**D1**, 50 mM **A1**, and 100 μM ATA-OA. Color use of the lines is consistent with that of the E^S labels. Source data are provided as a Source Data file.

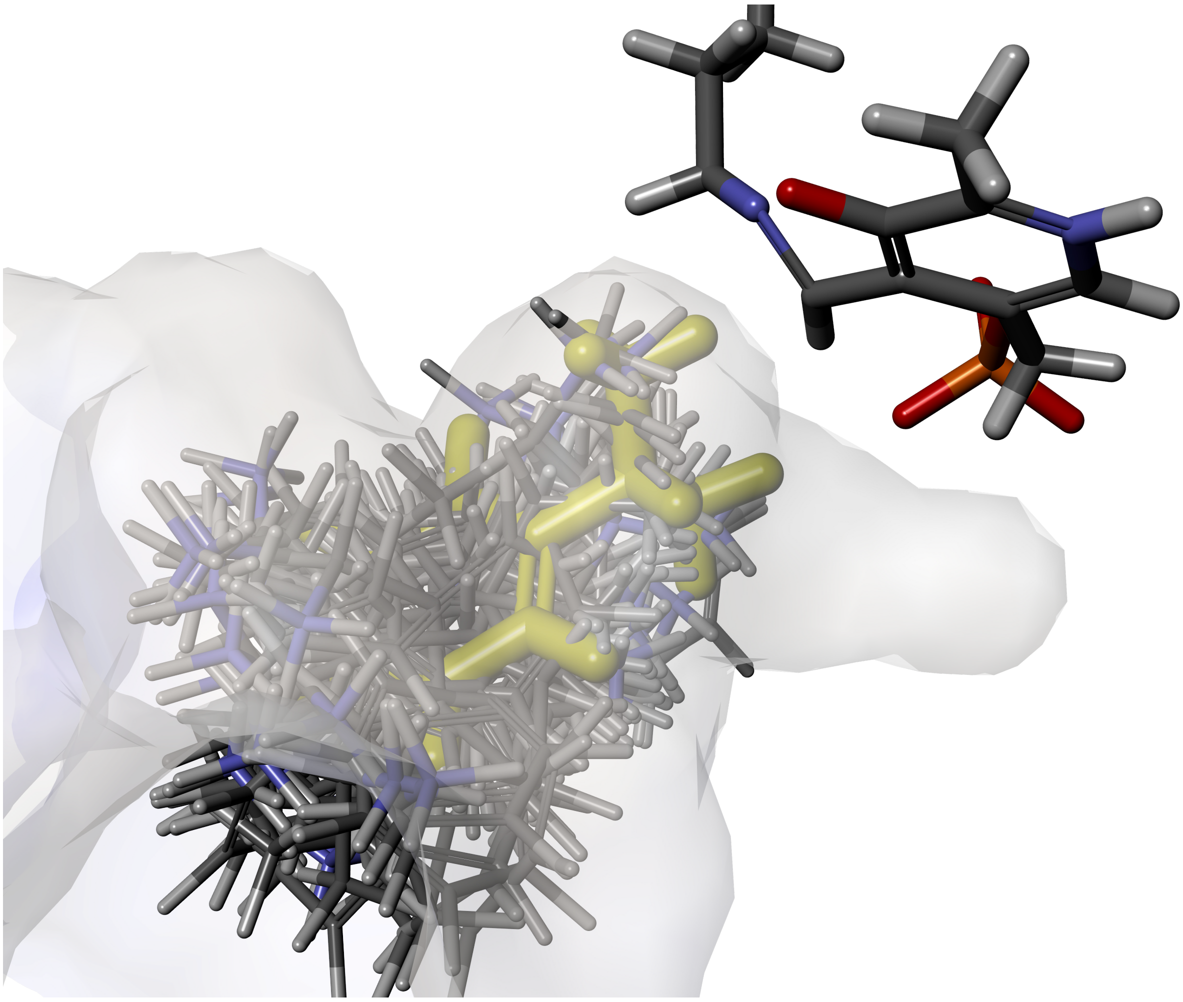

Supplementary Figure 9. All docking poses of *R*-**D1** in ATA-OA. Thick yellow sticks represent the top docking pose.

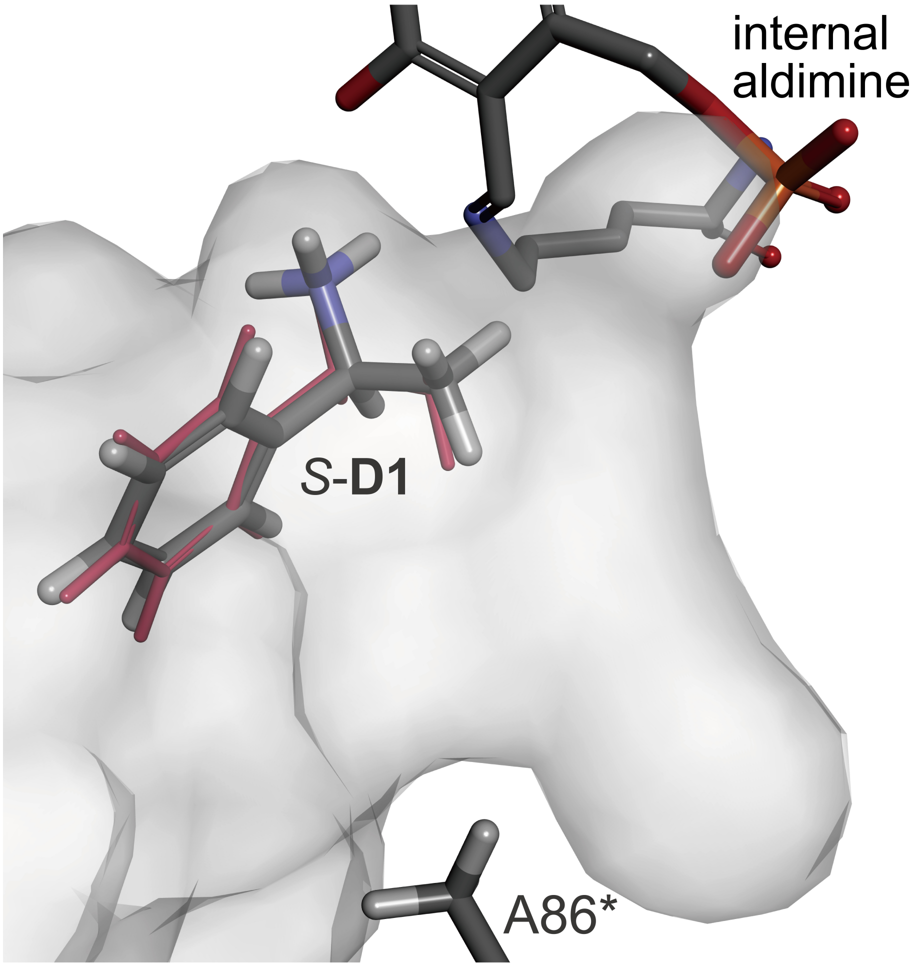

Supplementary Figure 10. The top docking pose of *S*-**D1** (thick sticks) in the F86*A mutant relative to that (thin magenta sticks) in the wild type.

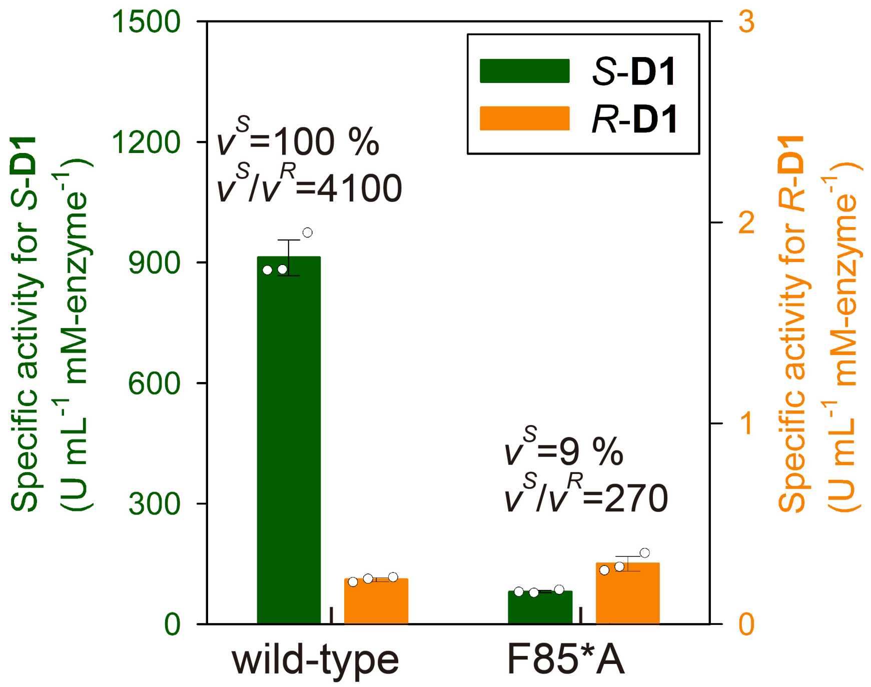

Supplementary Figure 11. Reduced stereoselectivity of ATA-PD for **D1** caused by the F85*A mutation. Note a 500-fold difference in scale between the left and right y-axis. Source data are provided as a Source Data file. Data are mean values of triplicate experiments with error bars indicating the s. d. (n = 3).

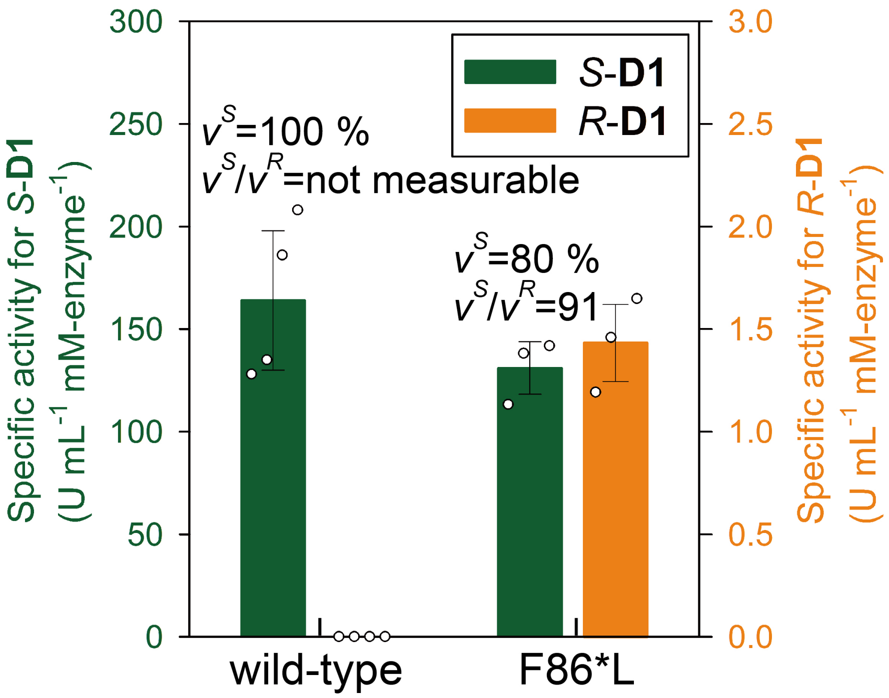

Supplementary Figure 12. Activities of the F86*L mutant of ATA-OA for *S*-**D1** and *R*-**D1** in comparison with those of the wild type. Note a hundred-fold difference in scale between the left and right y-axis. Source data are provided as a Source Data file. Data for wild-type are mean values of quadruplicate experiments with error bars indicating the s. d. (n = 4). Data for F86*L are mean values of triplicate experiments with error bars indicating the s. d. (n = 3).

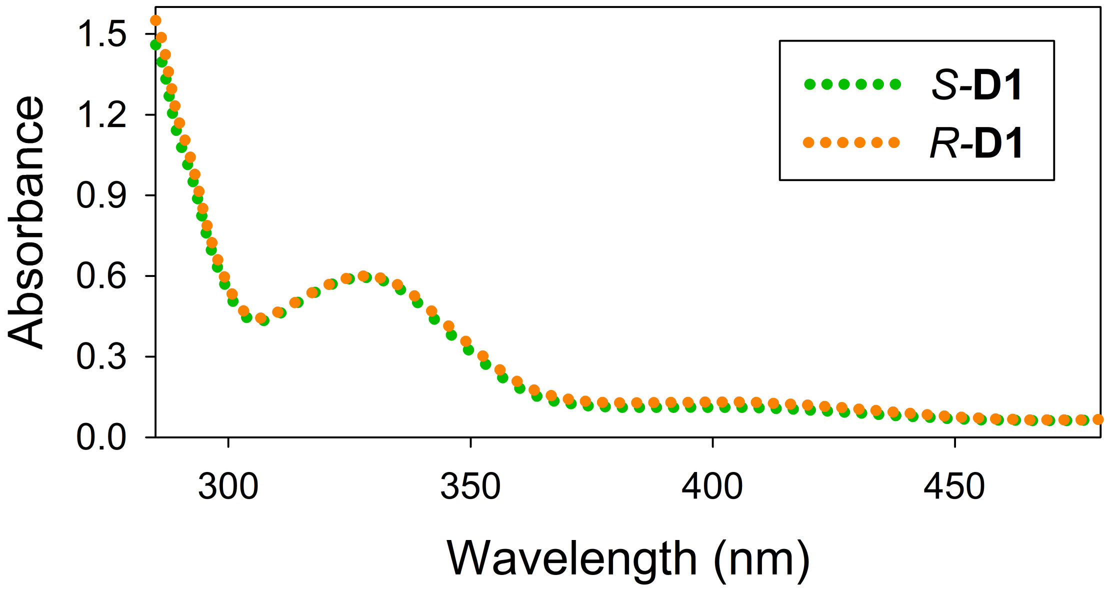

Supplementary Figure 13. Wavelength scanning of AR-OA equilibrated with *S*-**D1** or *R*-**D1**. Purified AR-OA (20 μM) was mixed with each enantiomer of **D1** (10 mM), followed by incubation at RT for 2 h before the spectral measurement. Source data are provided as a Source Data file.

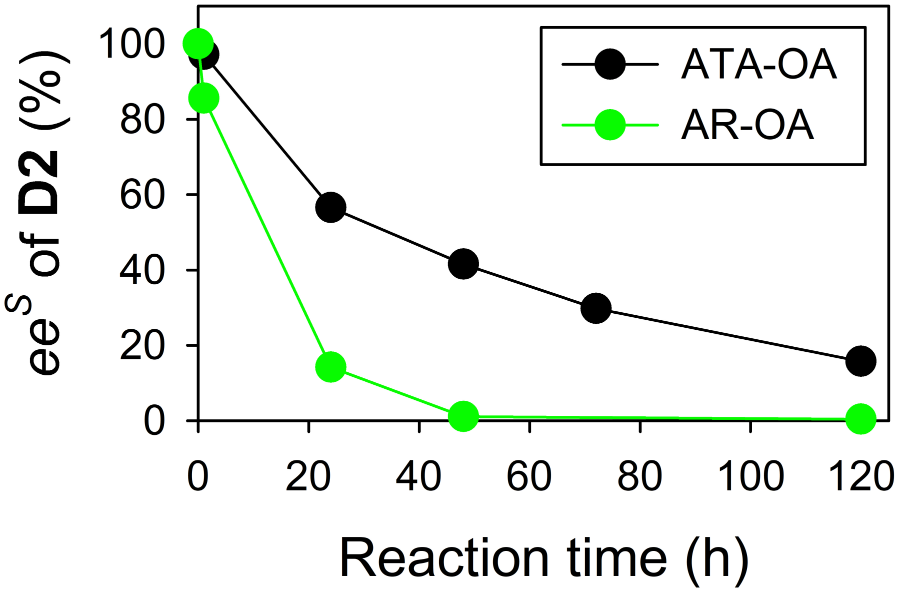

Supplementary Figure 14. Racemization of *S*-**D2** by AR-OA in comparison with that by ATA-OA. The AR reactions were carried out at 50 mM *S*-**D2**, 50 mM **A2**, and 0.5 mM PLP, and 100 µM enzyme. The ee^S profile for the ATA-OA reaction was taken from Fig. 3d. Source data are provided as a Source Data file.

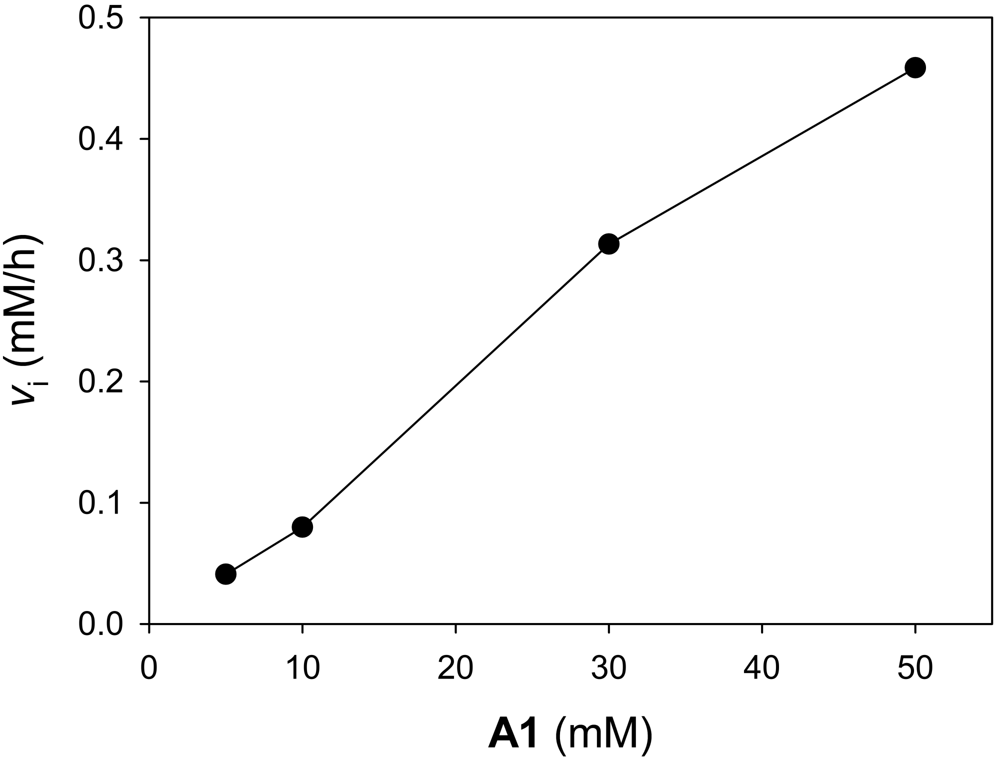

Supplementary Figure 15. Effect of the **A1** concentration on the initial rate of I^S/I^R by AR-OA. Reaction conditions were 100 mM *S*-**D1**, 5-50 mM **A1**, 1 mM PLP, and 50 μM AR-OA. Source data are provided as a Source Data file.

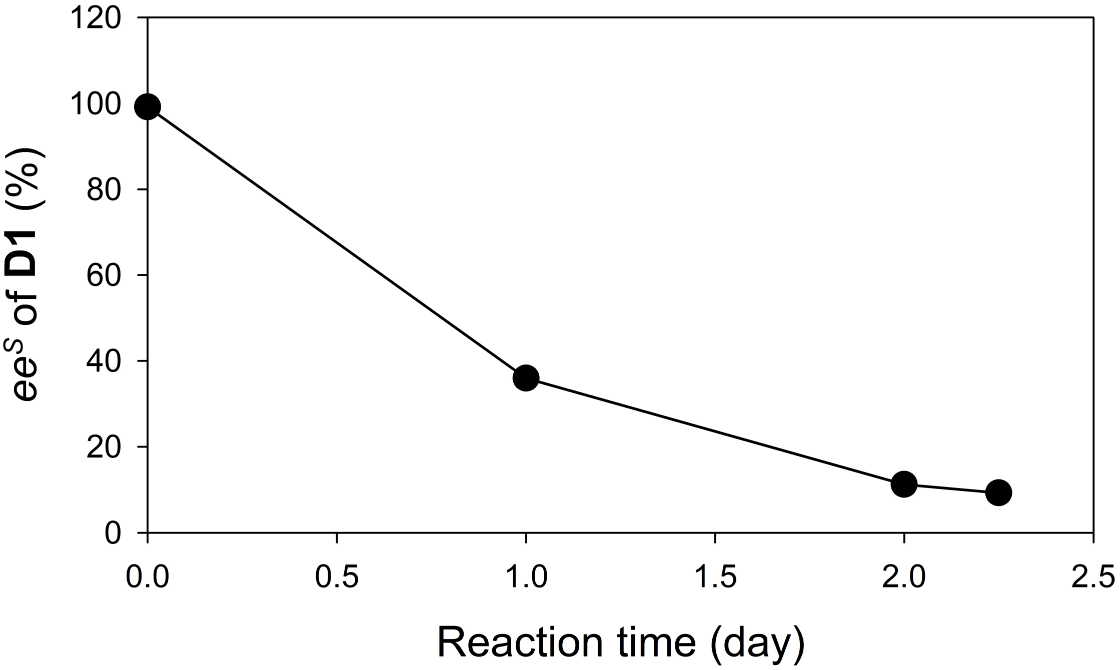

Supplementary Figure 16. Cosubstrate-free racemization of *S*-**D1** by AR-OA. Reaction conditions were 10 mM *S*-**D1**, 0.5 mM PLP, and 1.44 mM AR-OA. Source data are provided as a Source Data file.

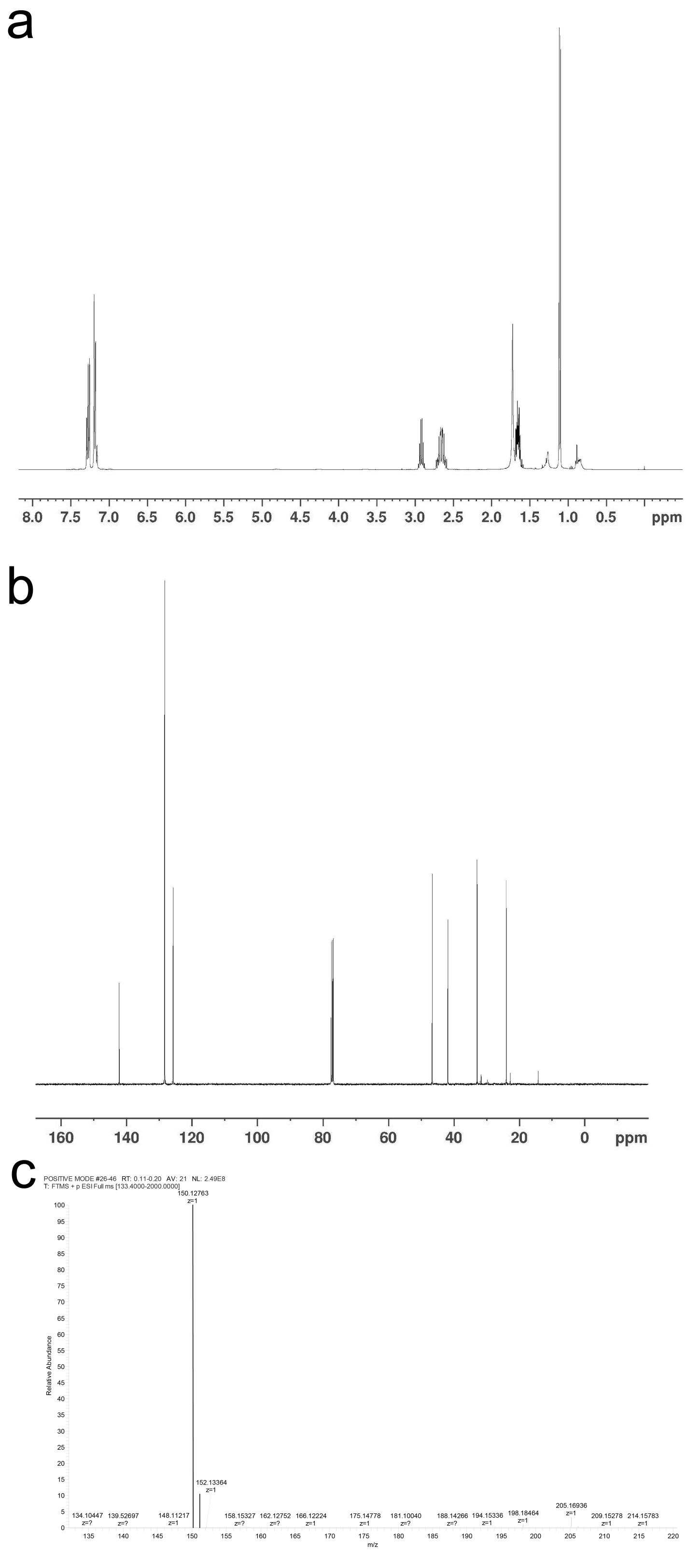

Figure 17. Structural characterizations of the isolated *rac*-**D5**. **a**, ^{1}H-NMR (400 MHz, $CDCl_3$): δ (ppm) 1.098-1.114 (d, 3H, CH_3), 1.619-1.684 (m, 2H, CH_2), 1.723 (s, 2H, NH_2), 2.584-2.720 (m, 2H, CH_2), 2.890-2.938 (m, 1H, CH) ,7.154-7.291 (m, 5H, CH). **b**, ^{13}C-NMR (400 MHz, $CDCl_3$): δ (ppm) 23.71, 32.83, 41.77, 46.55, 125.77, 128.04, 128.34, 142.26. **c**, HRMS (ESI): *m/z* $[M+H]^+$; calc'd for $C_{10}H_{15}N$, 150.1279; found, 150.1277.

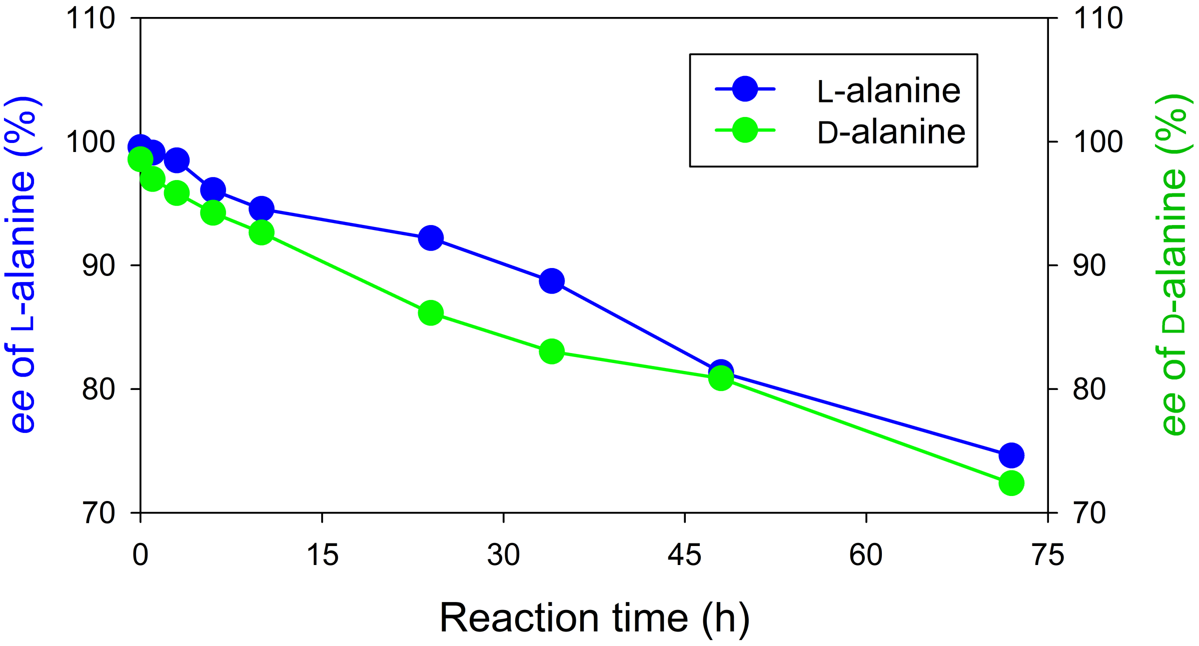

Supplementary Figure 18. Racemization of L- and D-alanine by the ATA-OA mutant carrying R417A and W58L substitutions. Reaction conditions were 10 mM L- or D-alanine, 10 mM pyruvate, 0.2 g/mL sodium azide, and 165 μM enzyme.

Supplementary References

1. Han, S.-W., Shin, J.-S. A facile method to determine intrinsic kinetic parameters of ω-transaminase displaying substrate inhibition. *J. Mol. Catal. B: Enzymatic* **133**, S500-S507 (2016).

2. Han, S.-W., Park, E.-S., Dong, J.-Y., Shin, J.-S. Mechanism-guided engineering of ω-transaminase to accelerate reductive amination of ketones. *Adv. Synth. Catal.* **357**,1732-1740 (2015).
