# Supplementary material for: Reprogramming biocatalytic futile cycles through computational engineering of stereochemical promiscuity to create an amine racemase
Source: Nat Commun. 2024 Jan 2;15:49. doi: 10.1038/s41467-023-44218-7 (PMC10761954; doi:10.1038/s41467-023-44218-7)
Supplement: Supplementary file 3 — Reporting Summary [file 41467_2023_44218_MOESM3_ESM.pdf]

Corresponding author(s): Jong-Shik Shin

Last updated by author(s): Dec 3, 2023

Reporting Summary

Nature Portfolio wishes to improve the reproducibility of the work that we publish. This form provides structure for consistency and transparency in reporting. For further information on Nature Portfolio policies, see our Editorial Policies and the Editorial Policy Checklist.

Statistics

For all statistical analyses, confirm that the following items are present in the figure legend, table legend, main text, or Methods section.

n/a	Confirmed	
☐	☒	The exact sample size (*n*) for each experimental group/condition, given as a discrete number and unit of measurement
☐	☒	A statement on whether measurements were taken from distinct samples or whether the same sample was measured repeatedly
☒	☐	The statistical test(s) used AND whether they are one- or two-sided<br>*Only common tests should be described solely by name; describe more complex techniques in the Methods section.*
☒	☐	A description of all covariates tested
☒	☐	A description of any assumptions or corrections, such as tests of normality and adjustment for multiple comparisons
☐	☒	A full description of the statistical parameters including central tendency (e.g. means) or other basic estimates (e.g. regression coefficient) AND variation (e.g. standard deviation) or associated estimates of uncertainty (e.g. confidence intervals)
☒	☐	For null hypothesis testing, the test statistic (e.g. *F*, *t*, *r*) with confidence intervals, effect sizes, degrees of freedom and *P* value noted<br>*Give P values as exact values whenever suitable.*
☒	☐	For Bayesian analysis, information on the choice of priors and Markov chain Monte Carlo settings
☒	☐	For hierarchical and complex designs, identification of the appropriate level for tests and full reporting of outcomes
☒	☐	Estimates of effect sizes (e.g. Cohen's *d*, Pearson's *r*), indicating how they were calculated

Our web collection on statistics for biologists contains articles on many of the points above.

Software and code

Policy information about availability of computer code

Data collection	UV absorbance data were collected by UVProbe (version 2.62). HPLC data were collected by EMPOWER Pro (version 2) and OpenLAB CDS ChemStation (version C.01.07) for Waters and Agilent HPLC, respectively.
Data analysis	Structural modeling, docking simulations, and preparation of structural figures were performed using Discovery Studio (version 4.5). Kinetic modeling was carried out using Mathematica (version 12). Microsoft Excel 365 (version 2310) was used for analyzing enzyme activities. Kinetic analysis and preparation of charts were carried out using Sigmaplot (version 12.5). ChemDraw (version 12.0) and Adobe illustrator (version 27.0) were used to draw chemical structures and to combine figures, respectively.

For manuscripts utilizing custom algorithms or software that are central to the research but not yet described in published literature, software must be made available to editors and reviewers. We strongly encourage code deposition in a community repository (e.g. GitHub). See the Nature Portfolio guidelines for submitting code & software for further information.

Data

Policy information about availability of data

All manuscripts must include a data availability statement. This statement should provide the following information, where applicable:

- Accession codes, unique identifiers, or web links for publicly available datasets
- A description of any restrictions on data availability
- For clinical datasets or third party data, please ensure that the statement adheres to our policy

The data supporting the findings of this study are included in the article and the Supplementary Information. The structural data of ATA-OA used in this study is from Protein Data Bank under accession code 5GHF (https://doi.org/10.2210/pdb5GHF/pdb). Source data are provided with this paper and have been deposited in figshare under https://doi.org/10.6084/m9.figshare.24217176.

Research involving human participants, their data, or biological material

Policy information about studies with human participants or human data. See also policy information about sex, gender (identity/presentation), and sexual orientation and race, ethnicity and racism.

Reporting on sex and gender	This research does not involve human participants.
Reporting on race, ethnicity, or other socially relevant groupings	This research does not involve human participants.
Population characteristics	This research does not involve human participants.
Recruitment	This research does not involve human participants.
Ethics oversight	This research does not involve human participants.

Note that full information on the approval of the study protocol must also be provided in the manuscript.

Field-specific reporting

Please select the one below that is the best fit for your research. If you are not sure, read the appropriate sections before making your selection.

☒ Life sciences ☐ Behavioural & social sciences ☐ Ecological, evolutionary & environmental sciences

For a reference copy of the document with all sections, see nature.com/documents/nr-reporting-summary-flat.pdf

Life sciences study design

All studies must disclose on these points even when the disclosure is negative.

Sample size	Sample size calculation was not performed for all experiments. Measurements of enzyme activities, kinetic constants, and enantioselectivities (figures 2e, 5c, 5d, and 6b) were conducted with a sample size of 3, because reliable calculation of the means and standard deviations of the measurement data were sufficient with three independent samples. In the case of the wild-type ATA-OA and its F86*A mutant (figure 4h), a sample size of 4 was used. A sample size of one was used for time-course analysis of reaction progress (figures 2a, 2b, 2c, 3d, 3e, 3f, 5e, 5f, 6a, and 6c) and UV absorbance (figure 2f), because a sample size of one sufficed to distinguish time-course changes of chemical concentrations and enzyme species. We used a sample size of one for the mathematical simulations of the reaction progresses (figures 3a, 3b, 3c, and 3d), because the results were the same across the repeated simulations. In the case of the preparative-scale reaction with S-D5, a sample size of one was used due to the limited availability of the substrate.
Data exclusions	We did not exclude any data unless the measurements were taken incorrectly by mistake.
Replication	Experiments were independently triplicated for measurements of enzyme activities, kinetic constants, and enantioselectivities (figures 2e, 5c, 5d, and 6b). In the case of the wild-type ATA-OA and its F86*A mutant (figure 4h), experiments were independently quadruplicated. Since a sample size of one was sufficient to distinguish time-course changes, experiments were conducted only once for the time-course analysis of reaction progress (figures 2a, 2b, 2c, 3d, 3e, 3f, 5e, 5f, 6a, and 6c) and UV absorbance (figure 2f). Mathematical simulations of the reaction progresses (figures 3a, 3b, 3c, and 3d) were performed once, because the results were consistent across the repeated simulations. The preparative-scale reaction with S-D5 was carried out only once due to the limited availability of the substrate.
Randomization	Bacterial colonies harboring the expression plasmids were randomly chosen from the agar plate for cell cultivation and protein expression. For the preparation of enzyme variants, transformants randomly selected from the agar plate were subjected to plasmid preparation and DNA sequencing. Randomization is not relevant to other experiments, including enzyme activity measurements, structural modeling, kinetic modeling, and racemization reactions.
Blinding	In this study, we performed enzyme assays, structural modeling, kinetic modeling, enzyme mutation, and biocatalytic reactions. To execute

Blinding	experiments based on a predetermined experimental design, we required knowledge of the chemicals and enzyme variants used for measurements and modeling. Therefore, blinding was not considered relevant to this study.

Reporting for specific materials, systems and methods

We require information from authors about some types of materials, experimental systems and methods used in many studies. Here, indicate whether each material, system or method listed is relevant to your study. If you are not sure if a list item applies to your research, read the appropriate section before selecting a response.

Materials & experimental systems

n/a	Involved in the study
☒	☐ Antibodies
☒	☐ Eukaryotic cell lines
☒	☐ Palaeontology and archaeology
☒	☐ Animals and other organisms
☒	☐ Clinical data
☒	☐ Dual use research of concern
☒	☐ Plants

Methods

n/a	Involved in the study
☒	☐ ChIP-seq
☒	☐ Flow cytometry
☒	☐ MRI-based neuroimaging
